# Use of a Sprayable Sex Pheromone Formulation in Landscape-Level Control of *Choristoneura fumiferana* Populations

**DOI:** 10.3390/insects13121175

**Published:** 2022-12-17

**Authors:** Lucas E. Roscoe, Wayne MacKinnon, Jacques Régnière, Glen Forbes, Matt Brophy, Rosanna Lamb

**Affiliations:** 1 Natural Resources Canada, Canadian Forest Service, Atlantic Forestry Centre, Fredericton, NB E3B 5P7, Canada; 2 Natural Resources Canada, Canadian Forest Service, Laurentian Forestry Centre, Quebec City, QC G1V 4C7, Canada

**Keywords:** mating disruption, Tortricidae, *Choristoneura*, pest management, forests

## Abstract

**Simple Summary:**

Mating disruption for insect pests can be an effective and environmentally sensible method. For landscape-level population management of forest insects, however, statistically rigorous experiments can be difficult to undertake. In 2021, we tested a new microencapsulated formulation (CONFOUND_SBW_) that was designed specifically for low density spruce budworm populations in New Brunswick, Canada, in a fully replicated experiment. While adult trap catch was reduced by 90% in treatment blocks, larval density and apparent fecundity were not significantly affected when compared to those in untreated control blocks. Although mating disruption remains a potentially useful tool in landscape-level forest pest management, CONFOUND_SBW_ is not effective against spruce budworm at low population densities when applied in accordance to label rates and volumes.

**Abstract:**

*Choristoneura fumiferana* (SBW) is a major defoliating pest of balsam fir and spruce in eastern North America. As part of an integrated management strategy for SBW, we evaluated the effectiveness of mating disruption as a landscape-level population control tactic. Using a sprayable formulation (CONFOUND_SBW_) containing a synthetic sex pheromone blend, we treated five 300 ha blocks in Northern New Brunswick with an aerially applied microencapsulated mixture. There were significant reductions in adult trap catches in treated blocks compared to untreated control blocks. Branch sampling in treated blocks showed uniform distribution of CONFOUND_SBW_ deposition throughout the blocks. Population densities following treatment were not significantly affected when compared to densities in control blocks, or prior to treatment. Analysis of egg:adult ratios indicates that no immigration events occurred within treatment or control blocks. The lack of population reduction following treatment strongly suggests that widespread application of CONFOUND_SBW_ at a rate of 50 g of active ingredient per hectare is not an effective tool in controlling SBW populations.

## 1. Introduction

Pheromone production and perception mediate various critical behaviors in lepidopterans, in particular the courtship sequence [1,2]. Females produce and emit a blend of compounds that stimulate upwind flight in conspecific males [3]. Perception by the male stimulates a ‘zig-zag’ flight around the pheromone plume that enables them to locate the female source [4,5,6]. Given the largely species-specific nature of the pheromone blend and its necessary effectiveness at stimulating upwind flight in males, pheromone exploitation is often an important aspect of an integrated pest management program [7,8,9]. For example, synthetic pheromone blends are often the basis of effective monitoring programs, particularly when the density of target populations is low. Pheromones may also be useful as population management tools, including both mass trapping and “attracticide” techniques [10,11]. Application of pheromones can hinder the ability of conspecifics to locate one another and mate, thus reducing realized fecundity and future population densities [12]. The mechanism of mating disruption may be one of several, including but not limited to: false-trail following, sensory fatigue, and female-source camouflage [13]. While the mechanism underlying successful pheromone-based mating disruption may vary between management programs, the benefits of this technique can be substantial [8]. Combined with its low-toxicity, lower likelihood of resistance development than is the case for wide-spectrum pesticides, and lack of non-target effects, mating disruption represented an important addition to many integrated pest management programs around the globe [10,14].

The principles of pheromone application to disrupt the courtship sequence of a target pest have long been postulated [15,16]. The potential of mating disruption as a management technique was first demonstrated in *Trichoplusia ni* (Hübner) where adult male trap catch was eliminated in blocks containing point sources emitting synthetic female pheromone [17]. Further utilization was demonstrated for other important agricultural pests including *Pectinomorpha gossypiella* (Saunders) (Lepidoptera: Gelechiidae) [18,19,20], and *Grapholitha molesta* (Busck) [21,22,23]. Though constraints on widespread use related to pest population suitability [24], and cost of production and application [25] were apparent, effectiveness equaled, and in some cases exceeded, that of insecticides and other treatment protocols [26,27,28].

A major forest insect pest for which mating disruption has been studied is the spruce budworm *Choristoneura fumiferana* Clemens (Lepidoptera: Tortricidae) (SBW). A defoliator of balsam fir *Abies balsamea* (L.) and spruces *Picea* spp. in North America, SBW outbreaks occur periodically every 30 to 40 years and may last 15 years in a given location [29]. In eastern Canada, landscape-level management of this species involves the application of the insect growth regulator Mimic^®^ (tebufenozide), and *Bacillus thurigiensis* var. *kurstaki*, an entomopathenogenic bacterium. Beginning in the 1970s, numerous experiments on the role of mating disruption in SBW management have been carried out [11]. Since then, important advancements in methods of application as well as the identification of the primary female pheromone components have occurred [30,31,32]. In 2007, the Hercon Disrupt Micro-Flakes^®^ SBW was commercially registered [33]. Several studies tested the effectiveness of this product and observed disruptive effects on male orientation and female mating success; however, statistical limitations associated with treatment replication due to logistical and financial restrictions existed [11]. Direct measurements of population dynamics pre- and post-treatment had also yet to be completed. These effects utilizing Hercon Disrupt Micro-Flakes^®^ SBW were recently quantified [34]. In addition to female mating success and pheromone trap capture, post-treatment population parameters including larval density and apparent fecundity in treatment sites were evaluated. These studies reported significant decreases in pheromone trap catch and mating success of caged females in treatment blocks; however, no significant effects on post-treatment larval and egg densities were found [34]. The lack of demographic effect was attributed to moth dispersal into relatively small (30–100 ha) treatment blocks, obscuring any tangible effects of pheromone treatment. Additionally, the deposition of pheromone on or near the forest floor due to the nature of the flakes themselves rather than in the tree canopies where the target adult moths are mostly found may impact treatment efficacy [12,33,35].

Recently, a sprayable microencapsulated mating disruption product for SBW (CONFOUND_SBW_, Registration number: 32730 PCP Act, Vantage, 707 Harco Drive Englewood, Ohio 45315, USA) was registered for use in Canada (Distributor: Andermatt Canada, 1350 Regent Street, Fredericton, New Brunswick E3C 2G6, Canada). Designed to adhere to branches after application, and thus remain in the tree canopy where most SBW mating occurs, this represents a new technology that may be more effective than previous SBW mating disruption formulations. Here, we conducted a fully replicated test of aerial application of this product over large (300 ha) blocks. We measured SBW populations at several stages before and after treatment in addition to pheromone trap catch. The goal of this study was to evaluate the effectiveness of this microencapsulated sex pheromone formulation (CONFOUND_SBW_) as a landscape-level population management tool that could be used to help mitigate the ecological and economic impacts of SBW.

## 2. Materials and Methods

### 2.1. Sites

**Figure 1:**
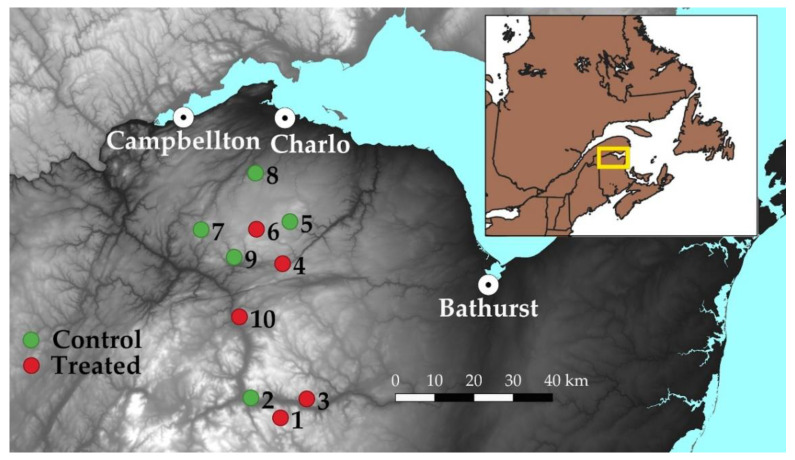
Location of treatment (red) and control (green) sites 1–10, used in the 2021 large-scale mating disruption field trial with CONFOUND_SBW_. Darker region: north-eastern New Brunswick, Canada; Paler region: south of the Gaspe Peninsula, Quebec, Canada.

Field trials were conducted in northern New Brunswick, Canada, south of Campbellton (48.00° N, −66.67° E) and west of Bathurst (47.62° N, −65.65° E) in summer 2021 (Figure 1). A total of ten 300 ha blocks (five treated and five untreated controls) were selected on parcels of Provincial Crown land located in areas with low SBW densities: 1 to 7 overwintering second-instar (L_2_) per 75-cm branch. In addition to low SBW population densities, sites were selected based on their proximity to the Early Intervention Strategy program treatments near the northern border of New Brunswick with Quebec [36] so that the risk of influx of adult SBW moths into test blocks was minimized. Each site contained a variety of stand types and tree species, with spruces (white, red, black) and balsam fir the dominant species. Sites also contained enough balsam fir near the centre of each block for repeated branch sampling with large sample sizes.

### 2.2. Population Measurements

Mid-crown branch samples (75 cm in length) were collected from dominant and co-dominant trees using pole pruners during winter 2021 (pre-trial) and fall 2021 (post-trial) from 100 randomly selected balsam fir trees at or near the centre of each block to estimate pre- and post-treatment densities of overwintering L_2_ larvae. Branch samples were washed in a 1% NaOH solution that dissolves the budworm’s hibernaculum, releasing the larvae. The loose contents of the wash were collected with a fine sieve and rinsed onto filter paper for counting using a dissection microscope [37]. Weather monitors were set up and operated at block centres between 21 June and 19 August to record temperature and relative humidity. Pupal sampling was completed between 6 and 9 July. Full pupae and empty pupal cases were extracted from the branches and counted to determine the local adult density in each block. Full pupae were reared until moths emerged and their numbers were added to the empty pupal case count to obtain an estimate of adult density in each block [38]. Egg mass sampling was conducted from 3 to 6 August. Egg masses were extracted from branches by examining all sides of all needles on branch samples. The number of eggs in each individual mass was counted under a binocular microscope. The egg counts were used to calculate the number of eggs laid per emerged adult, which in this highly mobile species provided an estimate of apparent fecundity, the sum of realized fecundity of locally emerged moths and eggs laid by immigrants, in each block [39].

Twenty of the randomly selected branches collected in fall of 2021 for post-treatment L_2_ densities were used to estimate the defoliation of current (2021) shoots. We used the Fettes method that consists of averaging the percent needle loss on 20 individual shoots from each branch [40].

### 2.3. Pheromone Monitoring

**Figure 2:**
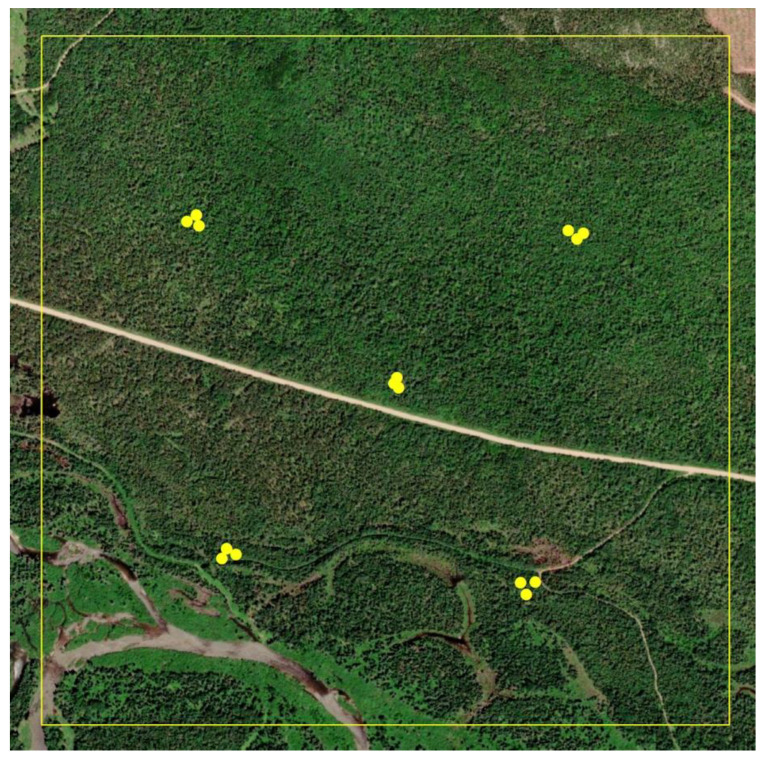
Placement of pheromone traps in 5 clusters of 3 traps each within 300 ha study Block 2, as example. Traps within clusters were spaced 30 m apart.

Fifteen (15) pheromone traps placed in five clusters of three traps were placed within each block. Traps were installed between 21 June and 25 June. We used the standard Multipher trap baited with a rubber septum lure loaded with 300 micrograms of 95E:5Z (E11)- and (Z11)-tetradecenals [41,42]. Traps in each cluster of three were spaced 30 m apart, located in the center of each block and in the center of each of the four quadrants (Figure 2).

Pheromone traps were checked and emptied immediately following pheromone treatment, and regularly thereafter during the weeks of 19–22 July, 26–29 July, and 16–19 August. Centre clusters were also checked during pupal sampling from 6 to 9 July and during egg mass sampling from 3 to 6 August.

### 2.4. Pheromone Application

CONFOUND_SBW_ containing 11% 95E:5Z (E,Z)-11-tetradecenal was applied in this experiment. This sprayable microencapsulated product mixture contained, in addition to CONFOUND_SBW_, guar gum as an adhesive agent to improve rain fastness, DayGlo^®^ fluorescent dye as a tracer, and water. The product was applied using rotary-winged aircraft equipped with Micronair AU5000 rotary atomizers at a rate of 50 g of active ingredient in a total application volume of 3 litres per hectare. Applications were timed to correspond with peak SBW pupal abundance which coincides with the first adult males being caught in the pheromone traps. A 25 m buffer was applied to all designated water bodies, including wetlands and streams.

**Figure 3:**
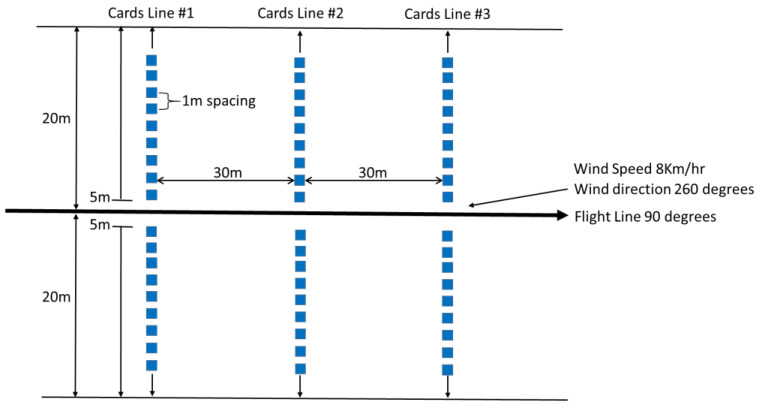
Configuration of the spray deposit calibration trial conducted at the Charlo airport, NB.

Prior to application, a spray calibration trial was conducted at the Charlo airport, NB (Figure 1) using a product mixture of guar gum, water, and blue dye. The calibration trial consisted of three transects of spray deposit cards (5 cm × 10 cm) spaced 1 m apart within transects. The transects were spaced 30 m apart, oriented at 90° of aircraft flight lines. Spray deposit cards were collected, and droplets were counted using a compound microscope. Wind during the calibration trial was 8 km/h at 260° (Figure 3).

Pheromone applications were completed in treatment blocks between 29 June and 3 July. Blocks 1, 3, and 10 were treated on 29 June, Blocks 4 and 6 on 2 July and 3 July, respectively, because of weather conditions. To evaluate spray deposit, three 150 m transects were established in each treatment block with a 20 cm branch tip sample taken every 5 m along each transect from balsam fir or spruce trees. Each transect consisted of 31 branch samples for a total of 93 per block. Transects were perpendicular to flight lines. Because of the presence of DayGlo^®^, spray droplets were easily observed under UV light. The presence/absence of spray droplets was recorded at the shoot level on each sample branch, separately for current and old distinguishing current and older shoots.

### 2.5. Statistical Analysis

To assess deposit, we calculated for each sample branch the proportions of current-year and older shoots with at least one spray droplet. A χ^2^ test was used to compare deposit between treated blocks at the branch level. Presence/absence of droplets was analysed by binomial logistic regression to determine the effects of shoot age class (current, older), host tree species (fir, spruce) and block nested within treatment on deposit.

To determine if pheromone treatment had a significant effect on captures in pheromone traps, a General Linear Model (GLM) analysis was performed on a box-cox transform of total catch in the three traps of each trap cluster *Y′* = (*Y*^0.25^ − 1), with treatment, trap position (center or edge) and block nested within treatment as factors. This transformation ensured normality of regression residuals (Anderson-Darling AD = 0.67, n = 50, *p* = 0.075).

Two measures were used to measure the efficacy of CONFOUND_SBW_ to disrupt the mating success of SBW: apparent fecundity (number of eggs laid per adult emerged on foliage), and population growth rate (pre-treatment L_2_ density compared to post-treatment L_2_ density). If treatment were to affect apparent fecundity, egg densities in treated blocks are expected to be lower than those in controls. Barring complete dispersal mixing of regional moth populations, we also expect a strong relationship between the density of adult populations (measured here as pupal cases found on foliage), and the corresponding egg population [39]. However, in the absence of any moth dispersal at the regional scale (in and out of blocks), we do not expect a relationship between apparent fecundity (eggs per adult) and pupal case density [39]. Thus, our analytical approach is one of covariance analysis, examining the effect of treatment on the relationships between pupal case density and egg density on apparent fecundity, using log transformed means (all logs herein are base 10).

## 3. Results

### 3.1. Spray Deposit

**Figure 4:**
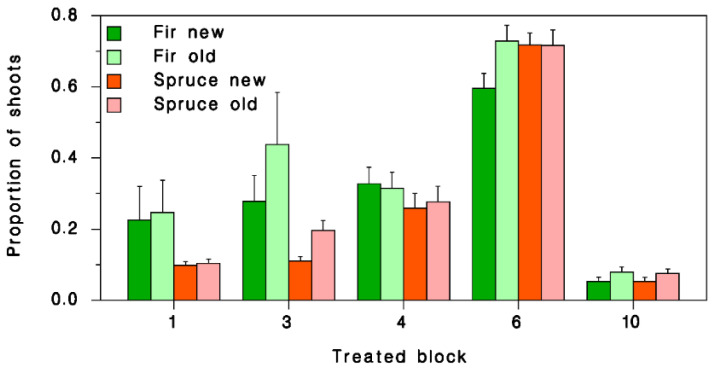
Mean (±SEM) proportion of shoots with at least one spray droplet on branches collected within blocks treated with CONFOUND_SBW_. Green: balsam fir; red: spruces; dark: current-year shoots; pale: older shoots.

The proportion of sample branches that had at least one spray droplet varied significantly between treated blocks (χ^2^ = 51.9, df = 4, *p* < 0.0001). This proportion was lowest in Block 10 (0.73). Deposit was higher and did not differ significantly among the other blocks (χ^2^ = 7.72, df = 3, *p* = 0.052). However, examined at the shoot level, there were significant differences in deposit between all blocks (highest in Block 6, lowest in Block 10), on the two host tree classes (higher on fir than on spruces) and shoot-age categories (higher on older shoots than on current-year shoots) (Table 1; Figure 4). We believe that low deposit in Block 10 resulted from spray drift.

### 3.2. Captures in Pheromone Traps

**Figure 5:**
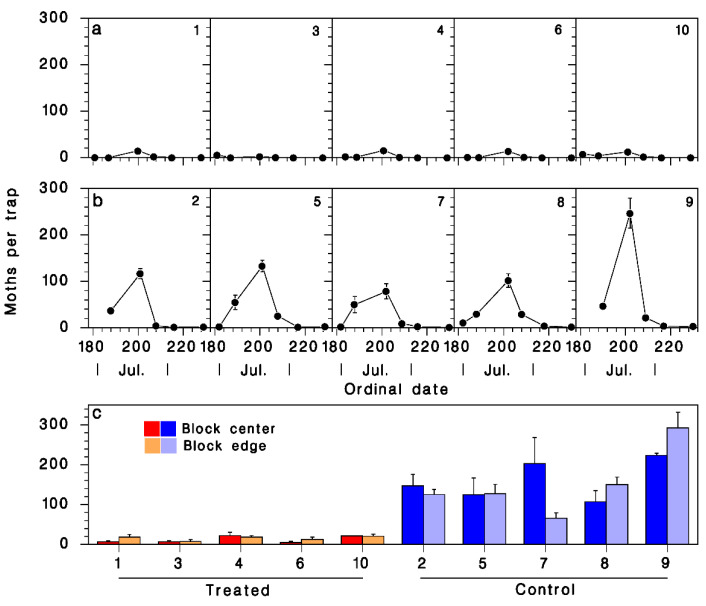
Mean (±SEM) adult SBW catch in pheromone traps. (**a**) In treated and (**b**) untreated blocks between June 30 and 19 August 2021. Block numbers in top right corners of panels. (**c**) Trap catch in center and at edges of blocks (Red: treated; blue: controls; dark: center; pale: edge).

Adult male SBW were caught in traps from 30 June to 3 August. No males were caught in any traps collected on or after 16 August (Figure 5a,b). The only significant effect on trap capture was treatment (Figure 5c; Table 2): traps in control blocks captured 153.9 ± 11.9 moths per trap, compared to only 14.6 ± 2.0 in treated blocks (a 90% trap catch shutdown).

### 3.3. Pheromone Treatment Efficacy on SBW Population Performance

**Figure 6:**
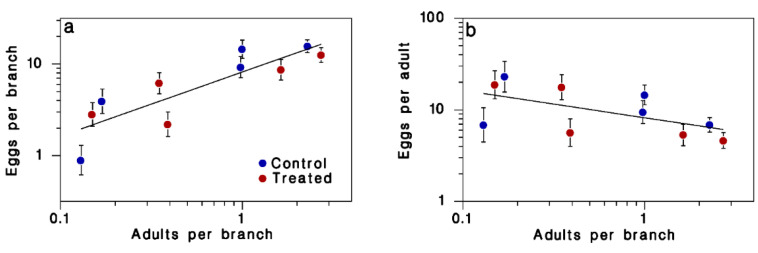
Relationship between pupal case (adult) density and (**a**) egg density or (**b**) apparent fecundity (eggs per adult). Means ± SEM; blue: control blocks; red: treated blocks. Line in (**a**) is Equation (1). Line in (**b**) has slope −0.299 ± 0.148, but this relationship did not reach significance (α = 0.05). Potential SBW fecundity is around 100 eggs/moth.

There was a significant relationship between egg and adult (pupal case) densities, but treatment had no significant effect on either the intercept or the slope of this relationship (Table 3). The regression model between egg density (*E*) and adult density (*A*), simplified to its single significant term, was:

(1) log(*E*) = (0.701 ± 0.148) log(*A*) + (0.914 ± 0.078)

(Figure 6a; R^2^ = 0.74; F = 21.29, df = 1,8, *p* = 0.002; test of residual normality: AD = 0.313, n = 10, *p* = 0.49).

There was no significant relationship between apparent fecundity (eggs per adult), and treatment or adult density (Table 3, Figure 6b; test of residual normality: AD = 0.252, n = 10, *p* = 0.656).

The data suggest very low realized fecundity (average: 11.3 ± 2.1 eggs per adult), which is approximately 10% of potential fecundity [39]. There is no evidence of a treatment effect on population reproduction (12.1 ± 3.1 eggs per adult among controls, 10.4 ± 3.2 among treated populations (F = 0.15, df = 1,8, *p* = 0.71). Thus, either there was considerable adult mortality or emigration from all blocks, or mating failure was quite common regardless of treatment. Mating failure is common at low SBW population densities [43].

Equation (12) in [39] proposes a method to estimate net egg laying by immigrant moths (*I*) and realized fecundity (*S*) of moths prior to emigration, using the non-linear regression model log(*E*) = log(*I* + *S M*) where *E* and *M* are egg and adult density. With this method, we estimated an immigration rate of *I* = 1.21 ± 0.79 eggs per branch, which was not significantly different from 0 (t = 1.55, df = 8, *p* = 0.08). Thus, our data suggest there was little if any immigration into our blocks. The corresponding estimate of realized fecundity of moths prior to emigration was only *S* = 6.67 ± 2.01 egg per adult, again suggesting that moths laid very few eggs in our blocks.

The relationship between apparent fecundity and adult density is expected to be negative as soon as moth dispersal plays an important role [39]. The negative slope in Figure 6b is not quite strong enough to have reached significance (−0.299 ± 0.148, F = 4.05, df = 1,8, *p* = 0.08). This again suggests negligible net immigration. Immigrants are expected to lay smaller egg masses than local moths, on average, because moths lay a significant portion of their eggs prior to take-off [44]. On average, egg masses contained 23.9 ± 8.6 eggs (SD). Eggs mass size was near-normally distributed (Anderson-Darling normality test AD = 0.58, n = 198, *p* = 0.13). The size of egg masses increased significantly with egg density on foliage (F = 10.3, df = 1,7, *p* = 0.015), and was not affected by treatment (F = 1.5, df = 1,7, *p* = 0.257). Thus, while egg density and apparent fecundity evidence suggest low immigration overall, egg mass size does point to some immigration into the lower density blocks.

**Figure 7:**
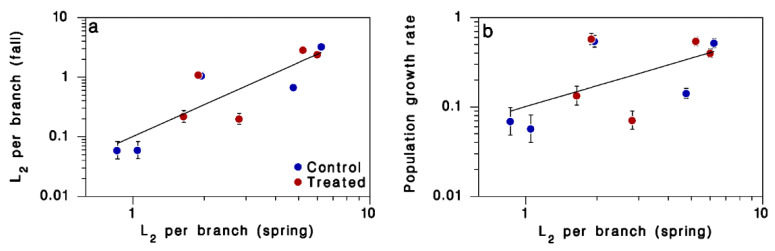
Relationship between spring L_2_ density and (**a**) fall L_2_ density or (**b**) population growth rate (from *L*_2*s*_ to *L*_2*f*_). Means ± SEM; blue: control blocks; red: treated blocks. Line in (**a**) is Equation (2). Line in (**b**) has slope 0.755 ± 0.385, but this relationship did not reach significance (α = 0.05).

Treatment had no significant effect on the relationship between density of L_2_ in the fall and that in the previous spring (Table 4; Figure 7a). The regression model between L_2_ in the fall (*L*_2f_) and L_2_ in the spring (*L*_2s_), simplified to the single significant term, was:

(2) log(*L*_2*f*_) = (1.775 ± 0.385) log(*L*_2*s*_) − (0.994 ± 0.197)

(Figure 7a; R^2^ = 0.73; F = 21.29, df = 1,8, *p* = 0.002; test of residual normality: AD = 0.205, n = 10, *p* = 0.821).

The population growth rate averaged 0.31 ± 0.07 (SEM), which is well below replacement. This is consistent with the very low apparent fecundity noted above. There was no significant effect of treatment on this value: 0.266 ± 0.109 among controls, compared to 0.345 ± 0.104 among treated populations. There was no relationship between population growth rate and the density of L_2_ in the spring (Table 4; Figure 7b, slope = 0.775 ± 0.385, F = 4.06, df = 1,8, *p* = 0.079).

## 4. Discussion

Numerous studies have evaluated the feasibility of mating disruption as a management tool for SBW (reviewed in [11]). Building upon the recommendations suggested from reviewing these trials and others more recent [34], we sought to conduct a large-scale evaluation of the latest mating disruption formulation (CONFOUND_SBW_). No treatment effects were found on population performance measures (apparent fecundity or growth rate); however, our results provide an important and novel contribution to the understanding of mating disruption for SBW using a microencapsulated formulation in future management programs.

Several considerations must be taken into account in the accurate evaluation of a mating-disruption trial [15]. Sufficient replication in any comparative study is clearly needed to distinguish block effects from treatment effects. In the 21 studies reviewed in [11], only three had >3 replicates. Another constraint likely associated with financial considerations is sampling effort required to accurately estimate population parameters. Equations for determining sample sizes required to achieve various levels of precision relative to mean spruce budworm population density are available [45], and sample size rapidly increases as densities being estimated drop. In many earlier mating-disruption trials, low replication and small sample sizes often led to inconclusive results [11]. That was not the case in the three trials reported in [34]. A major effort in the present study was directed towards maximizing statistical robustness in both replication and sample sizes. This allowed accurate measurements of L_2_, adult and egg densities. Those precise measurements allowed us to reach solid conclusions about efficacy.

A major consideration when initiating a mating disruption program is population densities within the treatment areas. Mating disruption effectiveness is maximized in areas of low population densities [12]. For example, the application of female pheromone is only recommended to target populations of *Cydia pomonella* (L.) (Lepidoptera: Tortricidae) not exceeding 1000 overwintering larvae per hectare [46]. Similar guidelines exist in protocols for mating disruption of *Lymantria dispar* L. (Lepidoptera: Lymantriidae), a rare example of successful control of a forest insect by mating disruption [47,48]. At close ranges, moths may rely on visual, tactile, and possibly auditory cues when locating conspecifics [12]. In areas of high density, where insects are clumped more closely together, moths are less reliant on long-range location using pheromones. Our pre-treatment sampling confirmed that SBW populations were low (<7 L_2_/branch), and thus satisfied this requirement.

An important component of SBW population dynamics is the dispersal of moths over the landscape [38,39]. While females rarely fly with a complete egg load [49], those that have deposited at least one egg mass will readily disperse [50]. This movement of moths, 60% females and 40% males caught during dispersal flight [44], can be a factor in the establishment of economically damaging populations at distances far away from their starting population. With respect to management programs, such dispersal in treated areas can mask any effects of the treatment [34]. Unless immigration into treatment blocks can somehow be avoided or prevented, an accurate measurement of efficacy is difficult to obtain. Indeed, such considerations are important in the installation of other programs, including but not limited to mating disruption for *C. pomonella* [46,51]. Two ways that such immigration could be minimized include the utilization of suitably large treatment blocks, and the local suppression of potential source populations in the surrounding area. Both ways guided our choice of size and location of study blocks. Based on comparisons of apparent fecundity, we concluded that little or no immigration occurred in any of our 10 study blocks. Furthermore, mean trap catches did not present large spikes that would suggest large immigration events [44].

A major impetus behind this test was the use of CONFOUND_SBW_ instead of the HERCON flakes. A potential advantage of the microencapsulated formulation versus the flake was the ability to apply and keep the pheromone product within the mid to upper crowns of treated trees. Following emergence, SBW adults are mostly found in the crowns of affected trees; it is here where most mating occurs [52]. In utilizing a microencapsulated formulation that adheres to branches within the crowns, the likelihood of maximizing exposure to synthetic pheromone is increased. Spray deposit analyses on branches collected from treatment blocks confirmed that pheromone was present within the canopies. This is further substantiated by the significant reductions in trap catches in the same blocks. The trap catch reductions of 90% were observed throughout the moth flight season, indicating that the pheromone components of the formulation had not degraded or been released too quickly after application. Such degradation in components or effectiveness would have reduced the likelihood of mating disruption. While these parameters confirm that pheromone was indeed present after application, no significant reductions in population performance were observed. It has been suggested that trap shutdown may need to exceed 95% for population reduction to be achieved with mating disruption [15]. Levels of 90% trap catch shutdowns were observed repeatedly in previous, well replicated trials with Hercon Disrupt Micro-Flakes^®^ SBW, with similarly disappointing results from a population management viewpoint [34].

The inclusion of all pheromone components specific to the target species in a synthetic mating disruption product has been suggested when developing an effective product [12]. CONFOUND_SBW_ includes the 95E:5Z (E11)- and (Z11)-tetradecenals primary SBW blend. Additional secondary pheromone *C. fumiferana* components were identified recently, and include (*Z*)-11-hexadecenal, (*Z*)-5-tricosene and (*Z,Z,Z*)-3,6,9-tricosatriene [53]. The inclusion of secondary components in mating disruption products could potentially increase product effectiveness, particularly in higher density infestations [44,54]. Presently, a microencapsulated formulation that includes all female SBW pheromones does not exist. While the development of such a product could be useful in future mating-disruption trials, financial considerations associated with product synthesis and application are currently prohibitive. The present cost to purchase and apply CONFOUND_SBW_ is about 4 × greater than currently registered insecticides used for protection against SBW. The inclusion of secondary components to the mating disruption product will only increase this price. Until the product can be formulated at a significantly lower price than current, any future landscape-level testing with additional components or higher active ingredient concentrations is unlikely.

## Figures and Tables

**Table 1:** Results of binomial logistic regression analysis of the proportion of shoots bearing at least one droplet of CONFOUND_SBW_ in treated Blocks 1, 3, 4, 6 and 10 (paired comparison odd ratios).

Paired Comparisons		Odds Ratio	95% Confidence Interval	
			Lower	Upper
Blocks				
3	1	0.9769	0.8863	1.0769
4	1	4.4844	4.1533	4.8420
6	1	15.863	14.433	17.436
10	1	0.4973	0.4484	0.5515
4	3	4.5903	4.1950	5.0228
6	3	16.238	14.612	18.045
10	3	0.5090	0.4542	0.5705
6	4	3.5375	3.2533	3.8465
10	4	0.1109	0.1008	0.1220
10	6	0.0313	0.0281	0.0350
Host species				
Spruce	Fir	0.8566	0.7942	0.9238
Shoot age				
Old	New	1.1857	1.1204	1.2548

**Table 2:** GLM analysis of total capture in pheromone traps in each cluster after a normalizing Box-Cox transformation.

Source	DF	Adj SS	Adj MS	F	*p* > F
Treatment	1	363.1	363.1	128.70	0.000
Trap position (Center/Edge)	1	0.705	0.705	0.25	0.621
Block (Treatment)	8	33.57	4.196	1.49	0.203
Treatment × Position	1	0.308	0.308	0.11	0.743
Block (Treatment) × Position	8	16.45	2.056	0.73	0.665
Error	30	84.65	2.822		
Total	49	723.7			

**Table 3:** Analysis of variance of the effect of log pupal case density log(*A*) and treatment on log egg density log(*E*) and log apparent fecundity log(*E*/*A*).

Source	DF	Adj SS	Adj MS	F	*p* > F
log(*E*) regression	3	1.18910	0.39637	7.75	0.017
Treatment	1	0.05678	0.05678	1.11	0.332
log(*A*)	1	0.89038	0.89038	17.42	0.006
log(*A*) × Treatment	1	0.06872	0.06872	1.34	0.290
Error	6	0.30669	0.05111		
Total	9	1.49579			
log(*E*/*A*) regression	3	0.28694	0.09565	1.87	0.235
Treatment	1	0.05678	0.05678	1.11	0.332
log(*A*)	1	0.01832	0.01832	0.36	0.571
log(*A*) × Treatment	1	0.06872	0.06872	1.34	0.290
Error	6	0.30669	0.05111		
Total	9	0.59363			

**Table 4:** Analysis of variance of the effect of L_2_ density in the spring log(*L*_2*s*_) and treatment on density of L_2_ in the following fall log(*L*_2*f*_) and population growth rate log(*L*_2*f*_/L_2*s*_).

Source	DF	Adj SS	Adj MS	F	*p* > F
log(*L*_2*f*_) regression	3	2.85312	0.95104	5.45	0.038
Treatment	1	0.01688	0.01688	0.10	0.766
log(*L*_2*s*_)	1	1.93502	1.93502	11.09	0.016
log(*L_2s_*) × Treatment	1	0.00776	0.00776	0.04	0.840
Error	6	1.04665	0.17444		
Total	9	3.89976			
log (*L*_2*f*_/*L*_2*s*_) regression	3	0.55897	0.186322	1.07	0.430
Treatment	1	0.01688	0.016882	0.10	0.766
log(*L*_2*s*_)	1	0.38808	0.388079	2.22	0.186
log(*L_2s_*) × Treatment	1	0.00776	0.007757	0.04	0.840
Error	6	1.04665	0.174442		
Total	9	1.60562			

## Data Availability

Data associated with this paper can be obtained at https://doi.org/10.23687/f53ee229-a482-499b-9d69-efb50b1bbfb9 (accessed on 14 November 2022).
